# Treatment With Endoscopic Ultrasound (EUS)-Guided Transesophageal Coil Embolization Without Sclerotherapy: A Novel Therapy for Giant Distal Esophageal Hemangioma

**DOI:** 10.7759/cureus.25303

**Published:** 2022-05-24

**Authors:** Khalid Al Shamousi, Zakariya AL-Naamani, Qasim Abbas, Said Al-Busafi, Haitham El Bingawi

**Affiliations:** 1 Department of Medicine, Sultan Qaboos University Hospital, Muscat, OMN

**Keywords:** sultan qaboos university, sultanate of oman, endoscopic ultrasound (eus), coil embolization, esophageal hemangioma

## Abstract

Giant esophageal hemangiomas are rare. The extent is usually in the mediastinum but may spread down to the upper abdomen. Such vascular pathology is hard to treat and typically to be resected along with the organs it is attached to. Here we report a novel way of treatment of giant distal esophageal hemangioma that was considered difficult for resection due to its anatomical spread. With endoscopic ultrasound (EUS) guidance, a few coils were placed in the hemangioma, which lead to stasis of blood and a significant reduction in the size of the lesion in over three months.

## Introduction

We are reporting a unique and rare case of a 54-year-old female who presented with symptomatic giant distal esophageal hemangioma and was treated with a novel endoscopic ultrasound (EUS)-guided coil placement technique. To the best of our knowledge, this is the first case reported for EUS coil embolization as a treatment for giant distal esophageal hemangioma.

## Case presentation

A 54-year-old female, with no past medical history, was admitted to an outside hospital with fever, atypical chest pain, and severe epigastric pain. Clinically she was not in heart failure, and no pericardial rub was found. She had elevated troponin of 310 ng/dl, and her ECG showed normal sinus rhythm with Q waves in the leads V1-V3. A transthoracic echocardiogram showed a thin rim of epicardial effusion, with a normal left ejection fraction of > 60%. A CT abdomen showed a large lobulated soft tissue density mass in the epigastrium measuring 8.3x10.1 cm. The bulk of the mass was at the gastroesophageal region encasing the aorta, celiac trunk, and superior mesenteric artery (SMA). She was diagnosed with myopericarditis and started on colchicine, aspirin, and atorvastatin.

**Figure 1:**
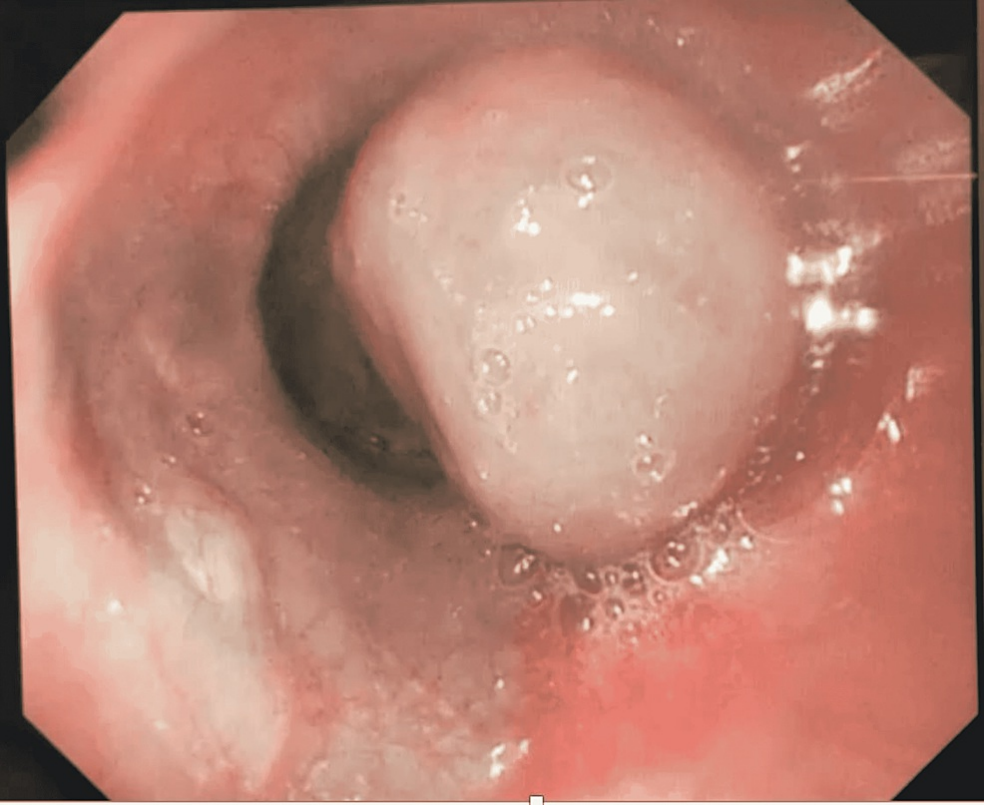
Endoscopic image of the 3 cm distal esophageal subepithelial mass

**Figure 2:**
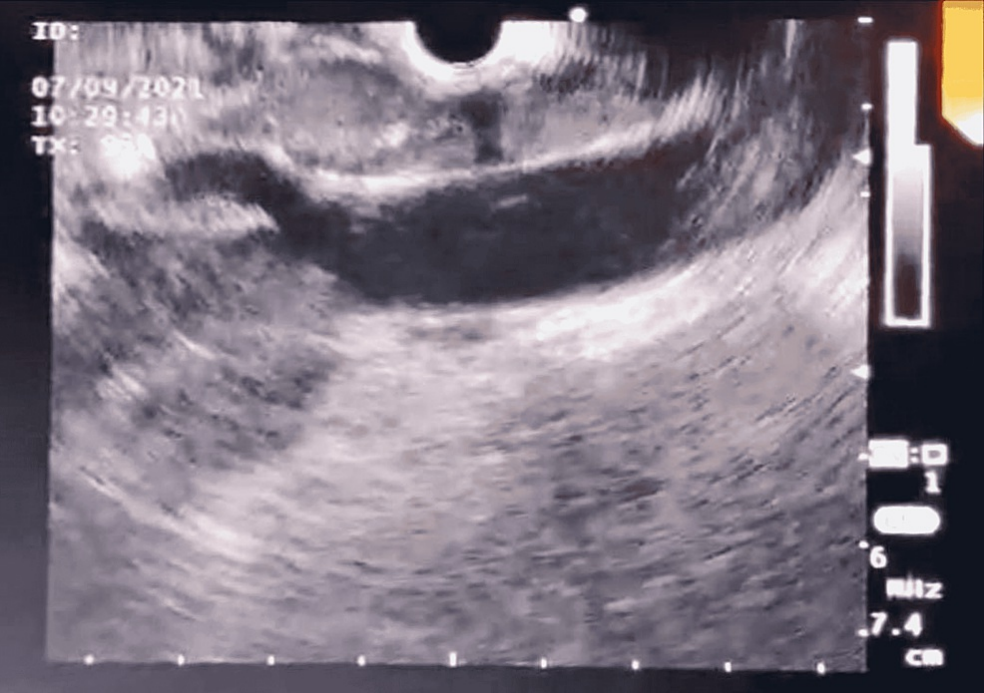
EUS image showing the vascular pathology encasing celiac artery take-off

**Figure 3:**
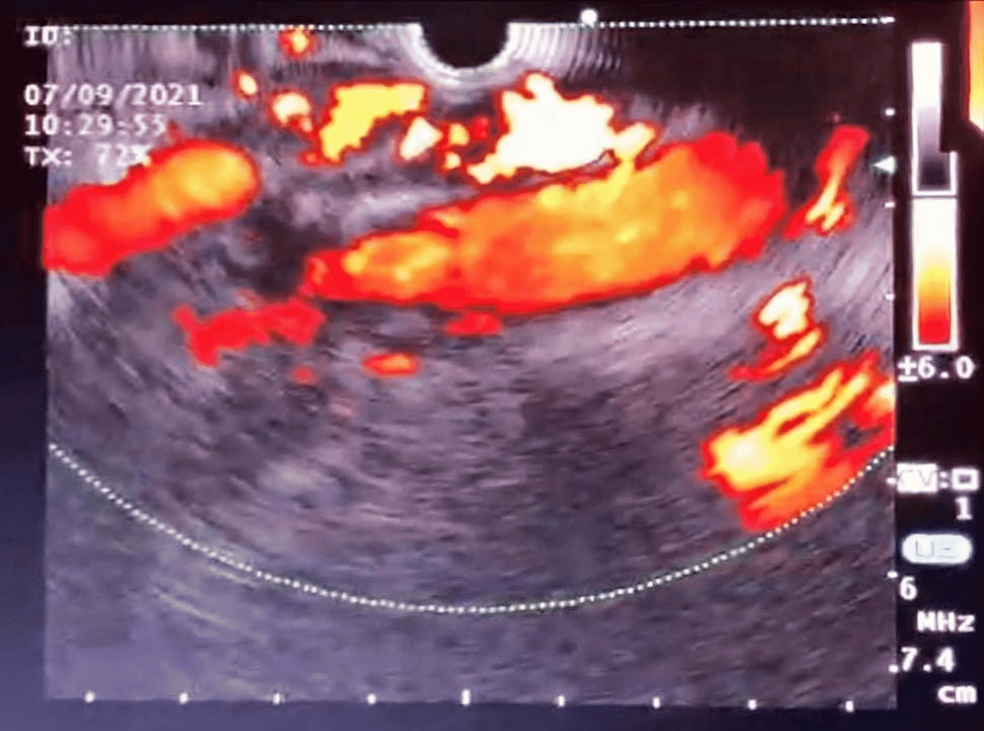
EUS image showing intense doppler signals of the vascular lesion around the celiac artery take-off

She was transferred to the Sultan Qaboos University Hospital, Muscat, Oman, in order to evaluate the epigastric soft tissue mass, which was seen on the CT scan. She underwent esophagogastroduodenoscopy (EGD) and oral EUS. The EGD detected a 3 cm, protruding, round esophageal sub-epithelial lesion at 30 cm from the incisors (Figure 1). On the EUS, the esophageal protruded lesion showed anechoic feature with dense doppler activity indicating a vascular network bulge (Figures 2, 3). Moreover, the lesion was engulfing the celiac artery take-off and superior mesenteric artery (SMA) with extension to the inferior pericardial area (Figures 2, 3). There were no detected enlarged lymph nodes in the EUS study.

**Figure 4:**
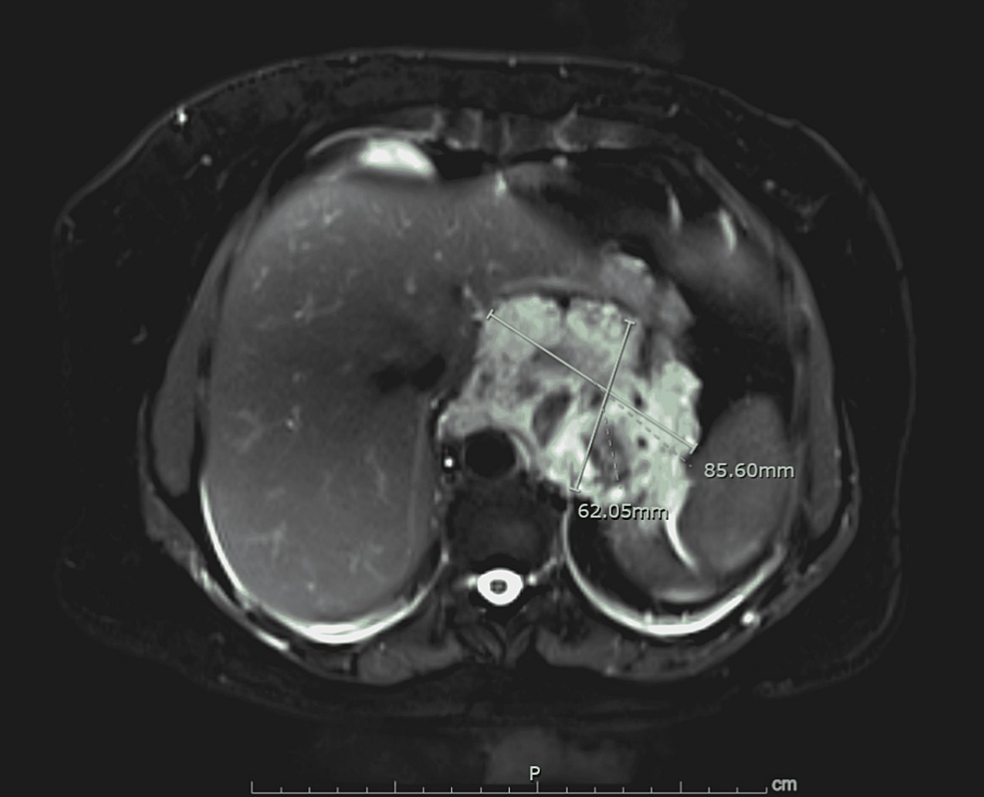
MRI abdomen showing low flow distal esophageal hemangioma

To better characterize the lesion, an MRI was done to classify the vascular pathology based on the speed of flow. The MRI showed an area of abnormal signal intensity in the retroperitoneum displacing the stomach slightly anteriorly and reaching up to the GE junction. Posteriorly, it was closely abutting the aorta. It extends into the retro-crural space. There was encasement of the celiac and superior mesenteric arteries origin. Multiple low attenuation areas were identified in this lesion, which is in keeping with numerous calcific foci (multiple calcified phleboliths). There was subtle delayed enhancement identified within it suggesting slow-flow vascular malformation. It appears intensely hyperintense on T2 weighted images (Figure 4). The diagnosis was made for massive distal esophageal hemangioma, which was infiltrating the lower esophagus and upper stomach.

The patient was hospitalized due to ongoing pulsating chest pain. A coronary CT scan did exclude any coronary artery disease. In order to decide the best therapy and due to the rarity of such a condition, we conducted a multidisciplinary team meeting that included vascular surgeons, upper gastrointestinal surgeons, interventional gastroenterologists, and interventional radiologists. The team concluded that the surgery is to be the last resort due to anatomical location and vascular encasement. A coil embolization to solidify the content of the hemangioma was felt to be the best option. EUS-guided route to place these coils were felt to be easier compared to the endovascular route due to the lack of identifiable feeder vessel.

**Figure 5:**
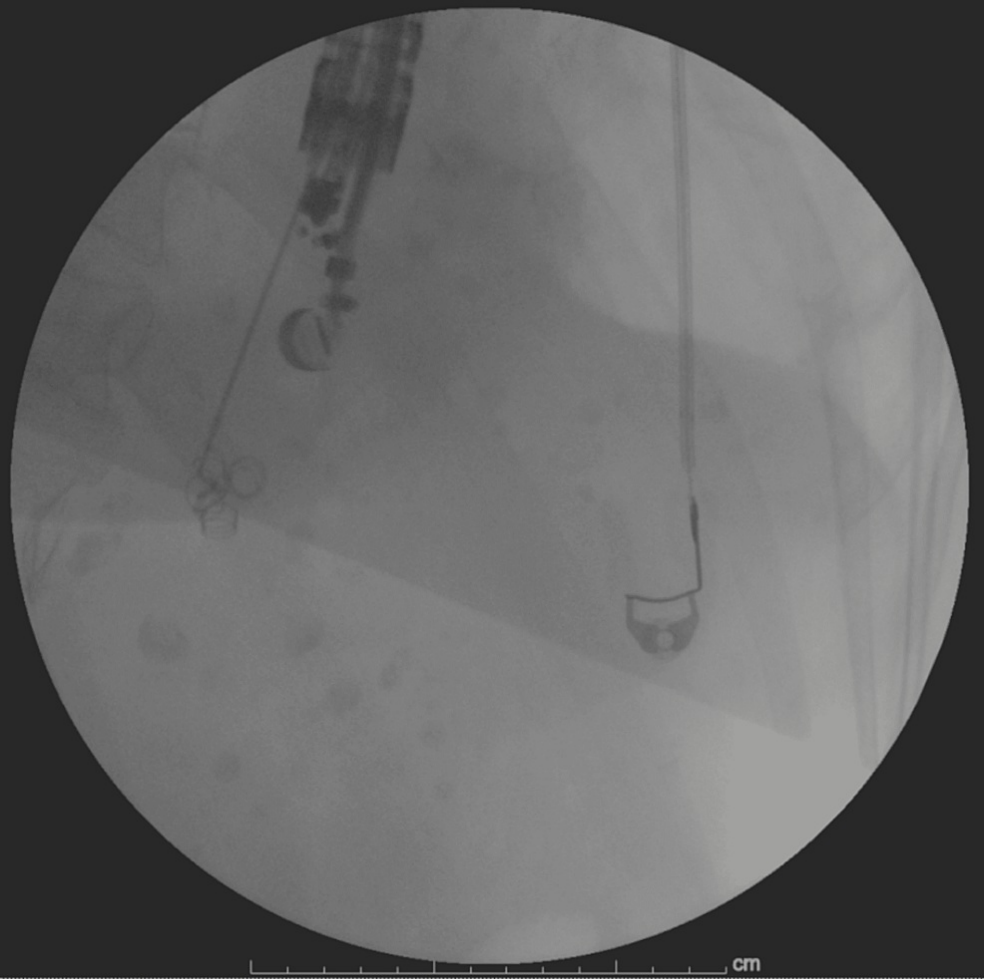
Fluoroscopic image of EUS-guided coil placement EUS: endoscopic ultrasound

The patient and her family agreed to the proposed plan. The patient underwent a EUS-guided coil embolization with the placement of five coils (Mreye® Embolization Coil 5mm, Cook Group Incorporated, Bloomington, Indiana, United States) using a 19G fine needle aspiration (FNA) needle through a transesophageal approach with fluoroscopic support. The doppler activity ceased after the coil's placement (Figure 5).

**Figure 6:**
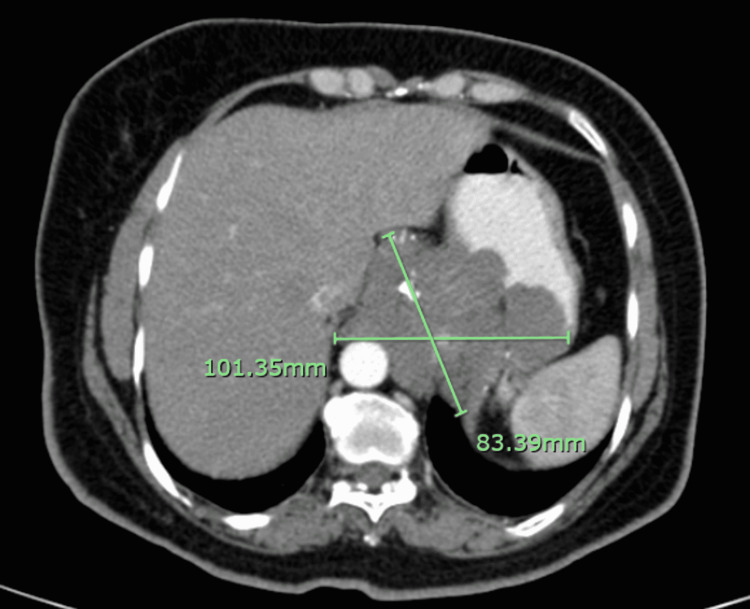
Computed tomography image showing the hemangioma before the coil embolization therapy

**Figure 7:**
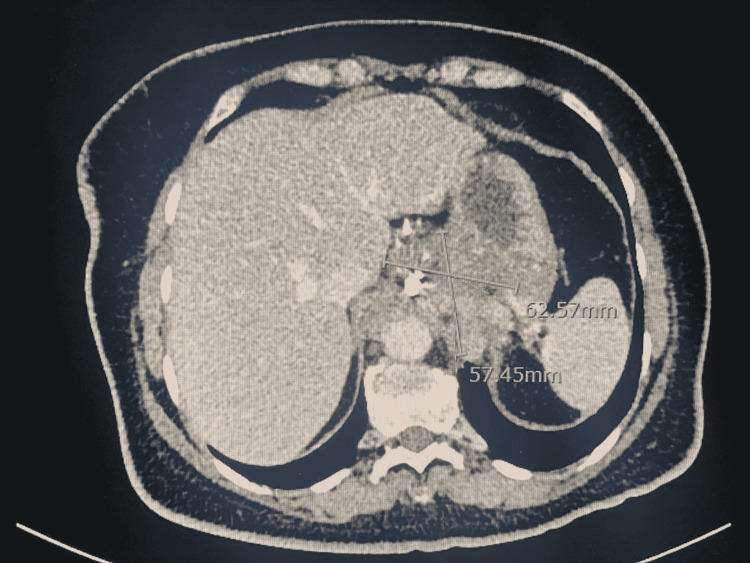
Computed tomography image showing the hemangioma after the coil embolization therapy

On day one post-procedure, the chest pain disappeared and the patient was discharged a few days later after a period of in-hospital observation and adequate rehabilitation. A follow-up CT scan of the chest was done three months after discharge and showed a significant reduction in the overall size of the hemangioma to 5.7x 6.2 cm. The patient remained asymptomatic since the placement of the coils (Figures 6, 7).

## Discussion

Giant esophageal cavernous hemangioma was described in 1976 by Feist, Talley, and Hunt. It was preoperatively 4 × 7 cm and pathologically 12.0×5.0×2.5 cm in size [1,2]. The presentation could be asymptomatic; however, most patients tend to exhibit symptoms including chest pain, dysphagia, and upper gastrointestinal bleeding that could be self-resolving or, at times, massive and life-threatening. Most reported cases were treated surgically with esophagectomy with a gastric pull-up via thoracotomy.

Our patient had a unique presentation of myopericarditis possibly due to mechanical rubbing between the giant hemangioma and the pericardium. Her symptoms evolved to pulsating chest pain and one episode of self-resolved minor upper gastrointestinal bleeding.

Endoscopic management for esophageal hemangiomas may include endoscopic or surgical approaches. Endoscopically, there have been reports of endoscopic mucosal resection, ablative argon therapy, or sclerotherapy, while surgical techniques usually involve distal esophagectomy with pull-up of the stomach [2]. A case of a small esophageal hemangioma (<0.5 cm) was reported to be successfully treated by repeated argon plasma coagulation [3]. Sclerotherapy can also be utilized for small lesions (<2 cm) with a lower risk of recurrence. A case report was published about sclerotherapy treatment for an 87-year-old woman with large (>20 cm ) esophageal cavernous hemangioma [4]. Another endoscopic technique used is endoscopic submucosal dissection (ESD), A large esophageal submucosal tumor (2.5 cm), which was suspected to be an esophageal hemangioma, was removed by ESD in a 50-year-old woman [5].

We applied a unique and novel therapy, which was used for the first time to treat a giant distal esophageal hemangioma. It was EUS-guided coil embolization without sclerotherapy. The technique aimed to solidify the blood and induce stasis to hinder the growth and possibly regress the size of the hemangioma. The intervention was successful in resolving all the symptoms of the patient immediately. As expected, the intervention led to a significant reduction of the hemangioma size as observed at the three-month follow-up based on CT measurement.

## Conclusions

A giant distal esophageal hemangioma is a truly challenging pathology to treat, especially if it is engulfing the surrounding vascular bed as in this case. EUS-guided coil embolization was used as a novel and minimally invasive technique. This was the first time, according to our knowledge that EUS-guided coil embolization was used to treat a giant distal esophageal hemangioma. The intervention was successful in immediately treating the symptoms. There was a significant size reduction of the hemangioma over the 90-day follow-up with CT scan.
